# Long-Term Efficacy of Neoadjuvant Concurrent Chemoradiotherapy for Potentially Resectable Advanced Siewert Type II and III Adenocarcinomas of the Esophagogastric Junction

**DOI:** 10.3389/fonc.2021.756440

**Published:** 2021-11-11

**Authors:** Yuan Tian, Jun Wang, Xueying Qiao, Jun Zhang, Yong Li, Liqiao Fan, Zhidong Zhang, Xuefeng Zhao, Bibo Tan, Dong Wang, Peigang Yang, Qun Zhao

**Affiliations:** ^1^ Third Surgery Department, The Fourth Hospital of Hebei Medical University, Shijiazhuang, China; ^2^ Department of Radiation, The Fourth Hospital of Hebei Medical University, Shijiazhuang, China

**Keywords:** neoadjuvant chemoradiotherapy, potentially resectable, advanced, Siewert II and III, adenocarcinoma of esophagogastric junction, treatment

## Abstract

**Background:**

Reports have shown that neoadjuvant concurrent chemoradiotherapy (nCRT) increases the R0 resection rate for patients with Siewert type II or III adenocarcinoma of the gastroesophageal junction (AEG). However, the long-term efficacy of nCRT for AEG patients remains unclear. In this multicenter study, we investigated the long-term results of AEG patients treated with nCRT.

**Methods:**

A total of 149 patients with potentially resectable advanced AEG (T3/4, Nany, M0) were randomly divided into two groups: the nCRT-treated group (treated group) (*n* = 76) and the surgery group (control group) (*n* = 73). The primary endpoint was disease-free survival (DFS), and the secondary outcome indexes included the R0 resection rate, HER-2 expression, tumor regression grade (TRG), objective response rate (ORR), disease control rate (DCR), overall survival (OS), and adverse events.

**Results:**

In the treated group, the overall therapeutic efficacy rate was 40.8%, and the pathological complete response (pCR) rate was 16.9%. The rates of patients who underwent R0 resection in the treated and control groups were 97.0% and 87.7%, respectively (*p* < 0.05). The toxic effects were mainly graded 1–2 in the treated group. The median DFS times in the treated and control groups were 33 and 27 months, respectively (*p* = 0.08), whereas the median OS times were 39 and 30 months, respectively (*p* = 0.01). The median DFS times of patients with positive and negative HER-2 expression in the treated group were 13 and 43 months, respectively (*p* = 0.01), and the median OS times were 27 and 41 months, respectively (*p* = 0.01).

**Conclusion:**

Surgery after nCRT improved the efficacy of treatment for AEG patients and thus provided a better prognosis.

**Clinical Trial Registration:**

The trial is registered with ClinicalTrials.gov (number NCT01962246).

## Introduction

In recent years, the incidence of adenocarcinoma of the esophagogastric junction (AEG) has been increasing worldwide (1–3). Due to the uniqueness of AEG, treatment for this condition has attracted increasing attention from scholars. Most clinicians believe that appropriate perioperative treatments should be used for AEG, and regarding this topic, an increasing number of researchers are trying preoperative concurrent chemoradiotherapy for AEG (4, 5). Undoubtedly, the Chemoradiotherapy for Oesophageal Cancer Followed by Surgery Study (CROSS) trial (6) is a milestone of preoperative concurrent chemoradiotherapy on AEG, but it still has some deficiencies, such as the inclusion of patients not only with AEG but also with lower esophageal cancer and squamous cell carcinoma and the inclusion of patients mostly in the early and middle stages. Trials on the treatment of Siewert type II and III AEGs are lacking. Siewert types II and III are representative of AEG, and the effect of neoadjuvant chemoradiotherapy (nCRT) is currently a research hotspot. The “Preoperative Concurrent Chemoradiotherapy for Potentially Resectable Adenocarcinoma of Esophagogastric Junction (NCT01962246)” trial conducted by our center has reported mid-term results (7) and verified a satisfactory surgical R0 resection rate and tolerable safety. The present study further summarizes the long-term follow-up data for this trial. We conclude that accurate clinical staging, target area delineation and radiation dose selection, efficacy evaluation, chemotherapy regimen and operation time after drug withdrawal, and perioperative nutritional support influence the treatment of Siewert type II and III AEGs. Based on these data, we attempted to provide a more reasonable solution for the preoperative treatment of AEG.

## Materials and Methods

The patient inclusion criteria for this study consisted of the following: (1) gastroscopy- and computed tomography (CT)-confirmed Siewert type II or III AEG with a long diameter of the primary tumor ≤8 cm prior to surgery; (2) American Joint Committee on Cancer (AJCC) 2010 classification of progressive gastric cancer before surgery (T3/4, Nany, M0) with no evidence of metastatic lesions in the liver, lung, brain, bone or other organs; (3) no prior antitumor therapy; (4) no contradictions to chemotherapy or surgery; (5) a Karnofsky Performance Scale (KPS) score >60 and an Eastern Cooperative Oncology Group (ECOG) score 0–2; and (6) informed consent obtained before enrollment in the study. All enrolled patients were randomly assigned to the concurrent chemoradiotherapy group or surgery group by using an interactive web-response system (IWRS). Patients were enrolled by authorized individuals who requested randomization with an IWRS integrated into the electronic case report forms (eCRF). Assignment to trial groups was completed on the server of the independent data management providers (Bioknow, Beijing, China) *via* a validated assignment program, which underlies strict access control. The randomization system assigned each patient a unique identification number and sent the researchers a message containing the results of the assignment.

### Regimen for Chemotherapy

The following XELOX regimen was applied for chemotherapy: capecitabine was administered at 1,000 mg/m^2^ twice daily for 14 days (day 1 to day 14); oxaliplatin was intravenously administered at 130 mg/m^2^ on day 1, and all subjects were treated for two cycles. Two cycles of chemotherapy were administered prior to surgery, and six cycles were administered after surgery. Eight cycles were administered after surgery in the control group.

### Regimen for Radiotherapy

(1) Radiotherapy planning CT scans were obtained with the patient in the supine position in a body mold to ensure setup reproducibility.

(2) CT simulation with intravenous (IV) contrast was performed to help guide the gross tumor volume (GTV) target, particularly for lymph nodes.

(3) The treating physicians utilized the following information to delineate active disease: barium meal, esophagoscopy/gastroscopy, and magnetic resonance imaging (MRI) scanning.

(4) Radiation targets included AEG, any perigastric extension, and lymph nodes (perigastric, celiac, portal hepatis, splenic hilar) with adequate margins. The standard GTV-t to clinical target volume (CTV)-t expansions were 2 cm in the superior-inferior direction and 0.8 cm laterally and anteroposteriorly. CTV-nd included CTV-nd and involved the field; 0.8–1.0 cm was added so that CTV+ 0.8–1.0 cm = planning target volume (PTV).

(5) Intensity-modulated radiation therapy (IMRT) was used and delivered by a linear accelerator as multiple shaped beams of 6 MV X-rays in five daily fractions of 1.8 Gy per week for 5 weeks (total PTV dose: 45 Gy).

### Determination of Therapeutic Efficacy

Therapeutic efficacy was determined according to the Response Evaluation Criteria in Solid Tumors (RECIST version 1.1). The response was made up of four classifications: complete response (CR), partial response (PR), stable disease (SD), and progressive disease (PD). The total efficacy [response rate (RR)] was calculated as the sum of CR and PR, and the tumor control rate was calculated as the sum of CR, PR, and SD. Tumor-node-metastasis (TNM) staging was performed according to the criteria developed by the AJCC (7th edition).

A tumor volume reduction rate of 12.5% was measured by CT as an effective threshold for evaluating neoadjuvant therapy (8).

Tumor volume reduction rate after chemotherapy = (tumor volume before chemotherapy − tumor volume after chemotherapy)/tumor volume before chemotherapy × 100%.

### Surgery

Laparoscopic exploration was performed 6–8 weeks after the end of concurrent chemoradiotherapy. Surgical treatment involved total gastrectomy and subsequent extended lymph node dissection (D2 resection). Reconstruction of the digestive tract involved Roux-en-Y esophagojejunostomy.

### Nutritional Support

The treated group started with 500 ml of the enteral nutrition (EN) suspension (total protein fiber, TPF) (Nutrison Fiber^®^), an oral nutrition supplementation (ONS) (500 ml per bottle containing energy 500 kcal, protein 20 g, fat 19.45 g, and carbohydrate 61.5 g), 7 days before surgery in addition to a routine preoperative diet (35 kcal/kg/day) according to dietary guidance. Patients in this group also received TPF 48 h after surgery *via* a nasojejunal tube placed during surgery. The feeding speed increased from the initial 30 ml/h according to the tolerance of the patients’ intestinal tracts. In general, nutritional support was shifted to the total EN 3–5 days after surgery, where patients were expected to start a semiliquid diet 4 days later. Consequently, the amounts of energy and protein were 25–30 kcal/kg/day and 1.0–1.5 g/kg/day, respectively, with the insufficient component supplemented with parenteral nutrition (9).

### Pathological Analysis

The pathological examination included the detection of tumor size, depth of invasion, number of metastatic lymph nodes, surgical margins, HER-2 expression, and tumor regression grade (TRG).

TRG was defined as follows: grade 0 (complete remission), no cancer cells; grade 1 (partial remission), single cells or a small group of cancer cells; grade 2 (low efficacy), residual cancer outgrown by fibrosis; and grade 3 (poor efficacy), minimum or no treatment effect with extensive residual cancer cells.

### Follow-Up

During the first year after treatment completion, patients received regular check-ups every 3 months. In the second year, regular follow-ups took place every 6 months and annually thereafter until 5 years after treatment. Additional interim visits were scheduled if complaints, such as renewed dysphagia and unexplained weight loss or pain, arose before the next scheduled visit. Diagnostic investigations were only undertaken as necessary measures during follow-up. No data on adverse events were collected beyond the initial report of this trial.

### Statistical Analysis

Statistical analysis was performed with SPSS version 19.0 software and GraphPad Prism version 7. Quantitative data were compared using the chi-square test. Qualitative data were compared using the *t*-test and are expressed as the mean ± standard deviation. The Kaplan–Meier method was used to calculate overall survival (OS) and disease-free survival (DFS). A *p*-value < 0.05 was considered statistically significant.

## Results

### Patient Data

**Figure 1:**
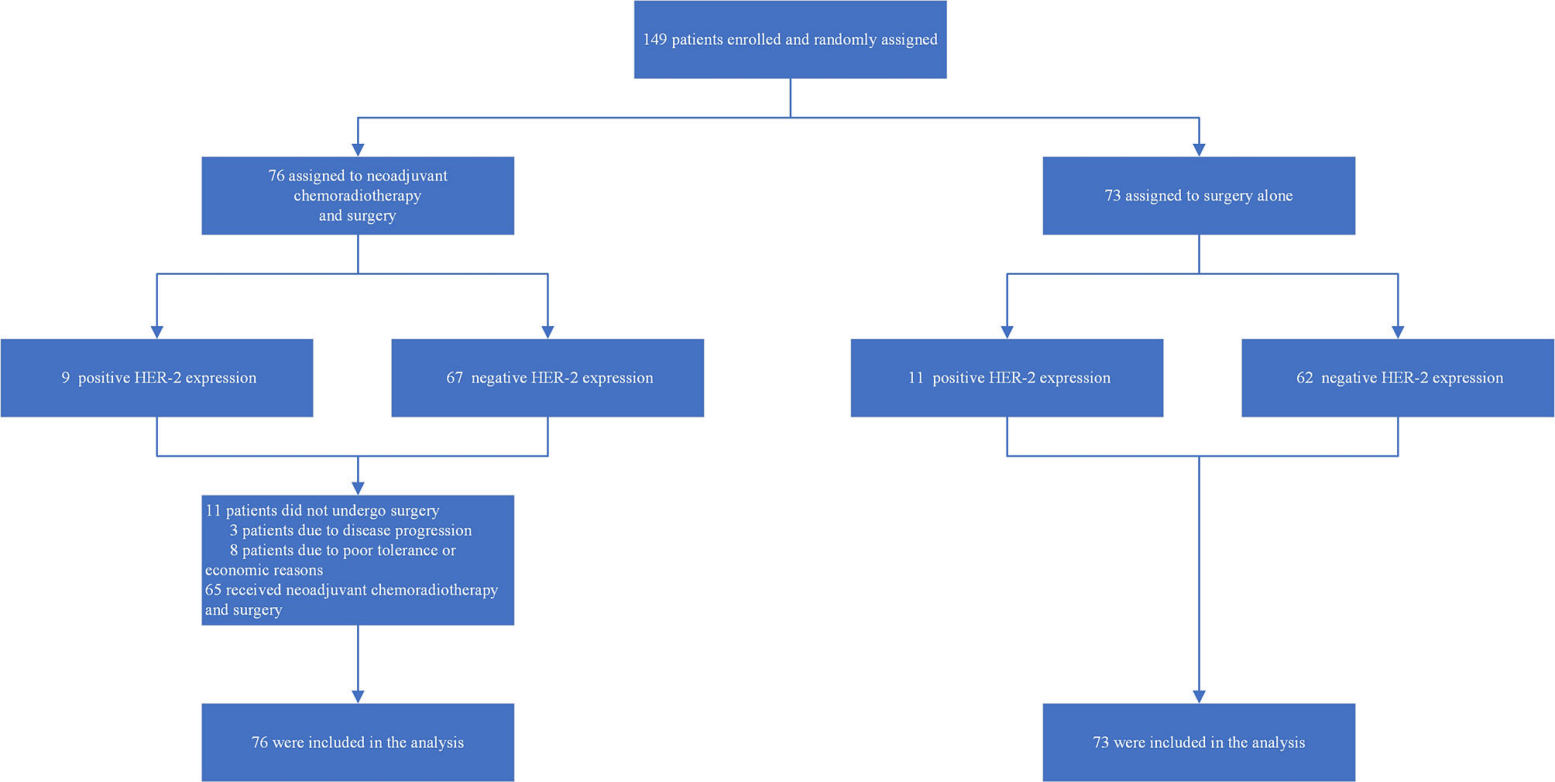
Trial profile.

A total of 149 patients with AEG who were admitted to the Fourth Hospital of Hebei Medical University between August 2012 and January 2016 were enrolled in this study. Patients were randomized at a 1:1 ratio using a stratified method (HER2 expression): a concurrent chemoradiotherapy group (*n* = 76) or a surgery group (*n* = 73). Patients in the concurrent chemoradiotherapy group (68 males and 8 females, median age 64 years, range: 43–75 years) received concurrent chemoradiotherapy and subsequent surgery. Patients in the surgery group (63 males and 10 females, median age 65 years, range: 42–74 years) were treated with surgery without chemoradiotherapy preoperatively. The general clinical characteristics of the patients in the two groups are shown in **Table 1**. After the end of concurrent chemoradiotherapy, 11 patients did not undergo surgery, 3 patients due to disease progression and 8 patients due to poor tolerance or economic reasons (**Figure 1**).

**Table 1:** General clinical characteristics of patients in the two groups.

	Concurrent chemoradiotherapy (*n* = 76)	Surgery alone (*n* = 73)	*p*
Age (years)	64 (43–75)	65 (42–74)	0.823
Sex [*n* (%)]			0.553
M	68 (89.5)	63 (86.3)	
F	8 (10.5)	10 (13.7)	
Vertical axis diameter of the tumor (cm)	4.6 (3–7)	4.4 (3–7)	0.757
HER2 expression			0.723
0	21 (27.6)	19 (26)	
1+	23 (30.3)	26 (35.6)	
2+ (FISH: negative)	23 (30.3)	17 (23.3)	
3+ (or FISH: positive)	9 (11.8)	11 (15.1)	
Clinical T stage [*n* (%)]			0.603
cT3	27 (35.5)	23 (31.5)	
cT4	49 (64.5)	50 (68.5)	
Clinical N stage [*n* (%)]			0.950
cN0	21 (27.6)	20 (27.4)	
cN1	20 (26.3)	18 (24.7)	
cN2	24 (31.6)	26 (35.6)	
cN3	11 (14.5)	9 (12.3)	
ECOG score [*n* (%)]			0.597
0	30 (39.5)	23 (31.5)	
1	36 (47.4)	39 (53.4)	
2	10 (13.1)	11 (15.1)	

### Clinical Efficacy

In the treated group, evaluation according to RECIST 1.1 revealed CR in 0 patients, PR in 31 patients, SD in 42 patients, and PD in 3 patients. The RR in the treated group was 40.8% (31/76), and the tumor control rate was 96.1% (73/76).

The tumor volume was 53.23 ± 21.57 cm^3^ before nCRT, and the tumor volume was 45.26 ± 22.39 cm^3^ after nCRT. Based on CT measurements of tumor volume reduction, the effective rate was 47.4%.

### Safety Evaluation

The hematologic toxic effects included leukopenia, neutropenia, anemia, thrombocytopenia, and abnormal liver function. The incidence of neutropenia in the treated group was greater than that in the control group, and the difference was statistically significant (65.8% *vs*. 38.4%, *p* = 0.034). The nonhematologic toxic effects included nausea, vomiting, diarrhea, constipation, hand–foot syndrome, and fatigue. These toxic effects were mainly graded 1–2. The incidence of nausea in the treated group was greater than that in the control group, and the difference was statistically significant (67.1% *vs*. 47.9%, *p* = 0.032). The incidence of fatigue in the treated group was greater than that in the control group, and the difference was statistically significant (61.8% *vs*. 39.7%, *p* = 0.022). The incidences of grade 3–4 hematologic and nonhematologic toxicities were low in the two groups, and the difference was not statistically significant (*p* > 0.05). Radiation gastritis/esophagitis and pneumonitis were unique to the treated group, with incidence rates of 43.4% and 13.2%, respectively, and these toxic effects were mainly grade 1–2 (**Table 2**).

**Table 2:** Toxic effects of concurrent chemoradiotherapy/adjuvant chemotherapy in the two groups [*n* (%)].

	Concurrent chemoradiotherapy: incidence rate (*n*, %)			Surgery alone: incidence rate (*n*, %)			*p*	
	Grade 1	Grade 2	Grade 3-4	Grade 1	Grade 2	Grade 3–4	Grade 1–4	Grade 3–4
Hematologic								
Anemia	30 (39.5)	7 (9.2)	3 (3.9)	23 (31.5)	7 (9.6)	1 (1.4)	0.340	0.611
Neutropenia	25 (32.8)	21 (27.6)	4 (5.3)	17 (23.3)	10 (13.7)	1 (1.4)	0.034	1.000
Thrombocytopenia	25 (32.8)	9 (11.8)	2 (2.6)	16 (21.9)	5 (6.8)	–	0.078	0.486
Liver dysfunction	17 (22.4)	2 (2.6)	–	13 (17.8)	3 (4.1)	–	0.374	–
Non-hematologic								
Nausea	28 (36.8)	19 (25)	4 (5.3)	18 (24.7)	13 (17.8)	4 (5.5)	0.032	1.000
Vomit	13 (17.1)	7 (9.2)	2 (2.6)	10 (13.7)	5 (6.8)	1 (1.4)	0.662	0.785
Diarrhea	9 (11.8)	6 (7.9)	4 (5.3)	9 (12.3)	4 (5.5)	3.6 (2/56)	0.374	0.673
Constipation	7 (9.2)	3 (3.9)	2 (2.6)	7 (9.6)	4 (5.5)	1 (1.4)	1.000	1.000
Hand–foot syndrome	14 (18.4)	6 (7.9)	4 (5.3)	13 (17.8)	8 (11)	1 (1.4)	1.000	0.354
Weak	40 (52.6)	7 (9.2)	–	26 (35.6)	3 (4.1)	–	0.022	–
Radiation gastritis/esophagitis	9 (11.8)	13 (17.1)	11 (14.5)	–	–	–	0.000	0.001
Radiation pneumonia	7 (9.2)	3 (3.9)	–	–	–	–	0.009	–

### Perioperative Complications

The incidence of wound infection, anastomotic bleeding, anastomotic leakage, abdominal infection, and intestinal obstruction was low, and the difference between groups was not statistically significant (*p* > 0.05). The incidence of pleural effusion in the treated group was higher than that in the control group, and this difference was statistically significant (23.08% *vs*. 6.85%, *p* = 0.007). The incidence of lung infection in the treated group was higher than that in the control group, and this difference was statistically significant (24.62% *vs*. 8.22%, *p* = 0.009). One patient in the treated group died during the perioperative period due to severe pulmonary infection (**Tables 3**, **4**). In the intention-to-treat analysis, the incidences of pleural effusion and pneumonia were also significantly different between the two groups (19.74% *vs*. 6.85%, *p* = 0.021; 21.05% *vs*. 8.22%, *p* = 0.027).

**Table 3:** Peri-operative complications in the two groups [*n* (%)].

	Concurrent chemoradiotherapy	Surgery alone	*p*
Incisional infection	4.62% (3/65)	2.74% (2/73)	0.556
Anastomotic bleeding	1.54% (1/65)	1.37% (1/73)	0.934
Anastomotic leakage	3.08% (2/65)	1.37% (1/73)	0.492
Abdominal infection	0% (0/65)	1.37% (1/73)	0.344
Intestinal obstruction	3.08% (2/65)	1.37% (1/73)	0.492
Pleural effusion	23.08% (15/65)	6.85% (5/73)	0.007
Pulmonary infection	24.62% (16/65)	8.22% (6/73)	0.009

**Table 4:** Grade III and above perioperative complications in the two groups [*n* (%)].

Complication	Concurrent chemoradiotherapy	Surgery alone	*p*
Grade III	5 (Pleural effusion)	2 (Pleural effusion)	
	1 (Anastomotic bleeding)		
Grade IV	1 (Pulmonary infection)	1 (Pulmonary infection)	
Grade V	1 (Pulmonary infection)	0	
Incidence of grade III and above	12.31% (8/65)	4.1% (3/73)	0.065

### Surgery and Pathological Evaluation

The R0 resection rates in the treated group and the control group were 97% (63/65) and 87.7% (64/73), respectively, and this difference was statistically significant (*χ* ^2^ = 4.012, *p* = 0.045). In the treated group, the pathological complete response (pCR) rate was 16.9% (11/65), and the total pathological response rate (grade 1 + grade 0) was 47.7% (31/65). The pathological lymph node metastasis rate and positivity rate were 43.1% and 3.9%, respectively, in the treated group and 76.7% and 20.9%, respectively, in the control group (**Table 5**). In the intention-to-treat analysis, there was no significant difference in the R0 resection rate between the two groups (86.3% *vs*. 87.7%, *p* = 0.806).

**Table 5:** Surgery and pathological evaluation in the two groups [*n* (%)].

	Concurrent chemoradiotherapy (%/*N*)	Surgery alone (%/*N*)	*p*
R0 resection rate	97 (63/65)	87.7 (64/73)	0.045
pCR rate	16.9 (11/65)		
TRG			
0	16.9 (11/65)		
1	30.8 (20/65)		
2	46.2 (30/65)		
3	6.1 (4/65)		
Lymph node metastasis rate	43.1 (28/65)	76.7 (56/73)	0.000
Lymph node positive rate	3.9 (73/1853)	20.9 (424/2031)	<0.05

### Follow-Up

**Figure 2:**
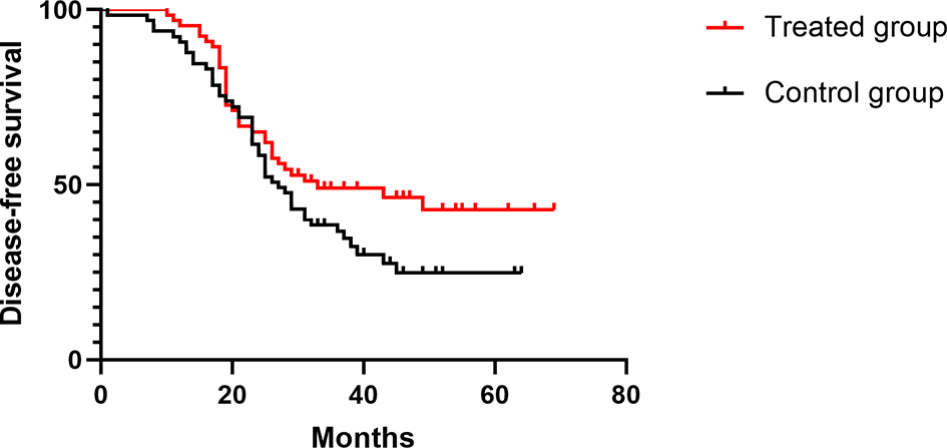
Comparison of DFS in the two groups.

**Figure 3:**
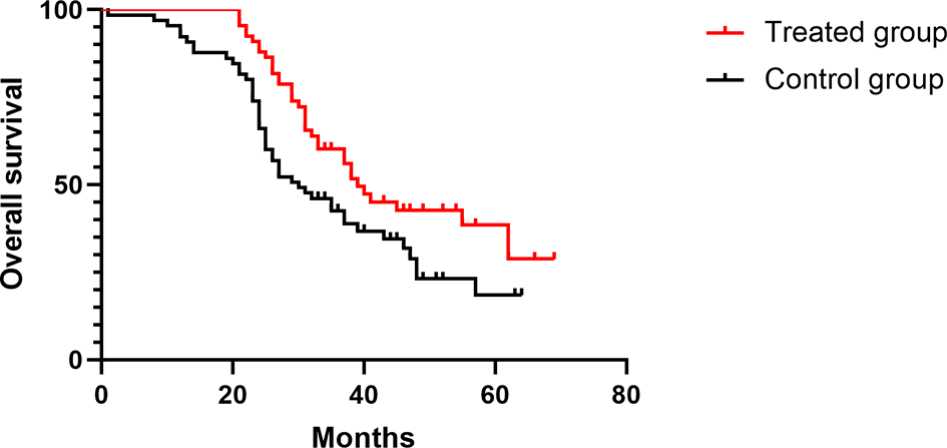
Comparison of OS in the two groups.

The median follow-up time was 52 months (27–77) in all patients, and the median DFS times in the treated group and the control group were 33 and 27 months, respectively (HR 0.68, [95% confidence interval (CI) 0.44–1.05], *p* = 0.08) (**Figure 2**). In the treated group, 30 patients had recurrence and metastasis, 8 patients had local recurrence, 27 patients had distant metastasis, and 5 patients had two or more recurrent metastases; therefore, the rate of total recurrence/distant metastases was 39.5% (30/76). In the control group, 39 patients had recurrence and metastasis, 20 patients had local recurrence, 26 patients had distant metastasis, and 7 patients had two or more recurrent metastases. Therefore, the rate of total recurrence/distant metastases was 53.4% (39/73) (**Table 6**). The median OS times were 39 and 30 months (HR 0.59, [95% CI 0.38–0.91], *p* = 0.01) (**Figure 3**), and the survival rates were 43.94% and 36.92% (*χ* ^2^ = 0.83, *p* = 0.362).

**Table 6:** Metastatic site in the two groups.

	Concurrent chemoradiotherapy (*N*/%)	Surgery alone (*N*/%)
Local recurrence		
Anastomotic/residual stomach	6 (7.9)	14 (19.1)
Regional tissue	2 (2.6)	6 (8.2)
Distant metastasis		
Lung	1 (1.3)	0
Liver	3 (3.9)	6 (8.2)
Bone	1 (1.3)	1 (1.4)
Brain	0	1 (1.4)
Peritoneum	17 (22.4)	15 (10.5)
Distant lymph node	5 (6.6)	3 (4.1)
Total recurrence/distant metastases	30 (39.5)	39 (53.4)

**Figure 4:**
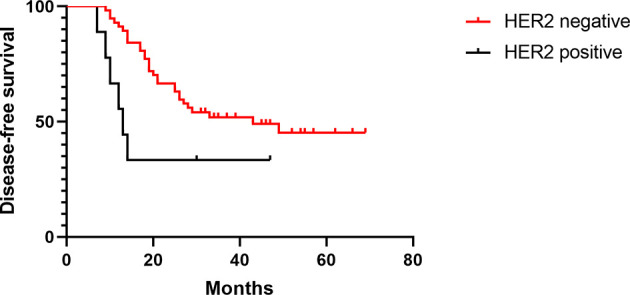
DFS of HER-2-positive and -negative patients in the test group.

**Figure 5:**
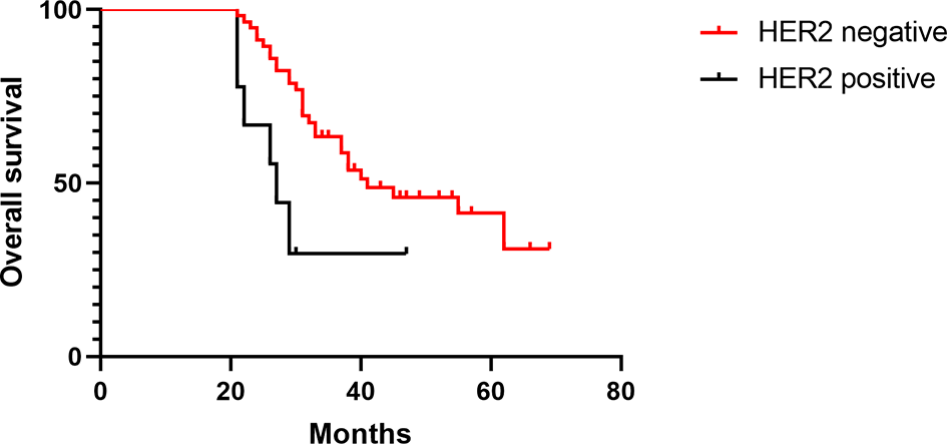
OS of HER-2-positive and -negative patients in the test group.

**Figure 6:**
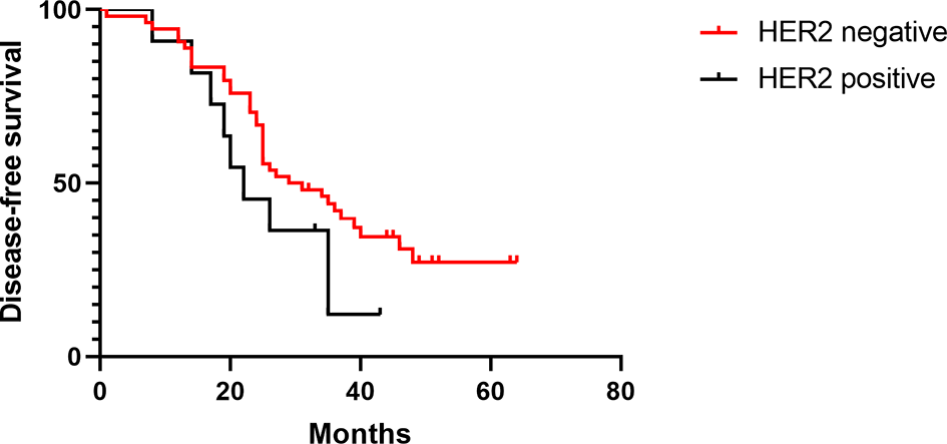
DFS of HER-2-positive and -negative patients in the control group.

**Figure 7:**
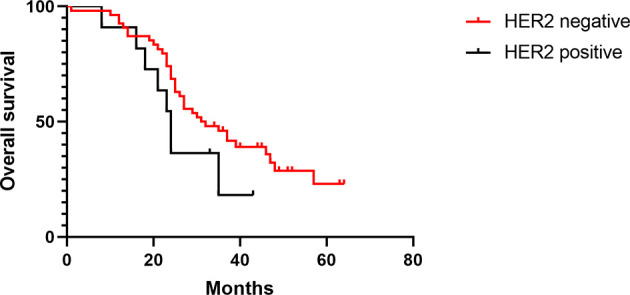
OS of HER-2-positive and -negative patients in the control group.

The median DFS times of patients with positive or negative HER-2 expression in the treated group were 13 and 43 months, respectively (HR 0.36, [95% CI 0.09–1.33], *p* = 0.01), and the median OS times were 27 and 41 months, respectively (HR 0.35, [95% CI 0.09–1.30], *p* = 0.01) (**Figures 4**, **5**). The median DFS times of patients with positive and negative HER-2 expression in the control group were 22 and 30 months, respectively (HR 0.57, [95% CI 0.24–1.39], *p* = 0.17), and the median OS times were 24 and 31.5 months, respectively (HR 0.59, [95% CI 0.23–1.49], *p* = 0.16) (**Figures 6**, **7**).

## Discussion

The efficacy of nCRT has been investigated in numerous clinical trials (10–12). Our results showed that patients with AEG who received nCRT benefitted more than those who received direct surgery according to the R0 resection rate and long-term survival. On this basis, we intended to seek a more efficient and safe treatment plan to prolong patient survival. Therefore, we performed preoperative nCRT on AEG patients according to the anatomical features of the esophagogastric junction.

In terms of the clinical and pathological evaluations, the effective rate was 40.8% in the treated group according to RECIST 1.1. At present, there are certain limitations associated with the clinical TNM staging system and RECIST, and the cTNM staging system differs from the pTNM staging system. RECIST can be used to evaluate solid tumors. However, for hollow organs, fluctuations in the degree of organ filling and the method used to select the longest diameter are obvious. Therefore, our center selected the tumor volume reduction rate after neoadjuvant treatment as the curative effect. The effective rate of nCRT was 47.4%, and although it was not completely consistent with the number of patients who received a pathological evaluation, it was similar to pathological efficiency (48.4%). However, tumor volume measurements, laparoscopic exploration and pathological HER-2 results could be used to supplement the clinical stage to select a more suitable treatment and predict prognosis.

There are significant differences in the delineation, dose, and range of radiotherapy for AEG (13–15). Although the European Organisation for Research and Treatment of Cancer (EORTC) elaborated on the delineation of preoperative radiotherapy target areas of AEG, there are some differences between the EORTC and National Comprehensive Cancer Network (NCCN) guidelines in the recommended high-risk lymph node prophylaxis areas. According to the literature reports, the difficulty of radiotherapy technology for AEG is mainly attributed to determining the boundary of the GTV, the reasonable expansion of the CTV, and the irradiation range of high-risk lymph node areas. Until recently, there have been few related studies and a lack of data on pathological results. There is no accepted standard for preoperative radiotherapy target area delineation. In this study, the water filling method was used for CT simulation, and the CTV range was mainly determined by the thickening of lesions displayed on enhanced CT images and the results of upper gastrointestinal angiography and gastroscopy. Standard GTV-t to CTV-t expansions were 2 cm in the superior–inferior direction and 0.8 cm laterally and anteroposteriorly. CTV-nd included CTV-nd and involved fields; 0.8–1.0 cm was added so that CTV + 0.8–1.0 cm = PTV. This radiotherapy program can achieve a better pCR rate, reduce the lymph node metastasis rate, and increase the R0 resection rate. The incidence of acute radiation inflammatory reactions is low, and the tolerance is good.

The pCR rate in this study was 16.9% (11/65), which was close to that of the PreOperative therapy in Esophagogastric adenocarcinoma Trial (POET) (14.3%) (5). A European study on the time interval between nCRT and surgery for esophageal or junctional cancer (16) showed that 906 (29%) of 3,091 patients achieved a pCR. In this study, we confirmed that the pCR rate was mainly related to the pathological type, duration of surgery or nCRT, and cT stage. An interval of ≥10 weeks for adenocarcinoma and ≥13 weeks for squamous cell carcinoma between nCRT and esophagectomy was associated with a higher probability of achieving pCR. The 30-day/in-hospital mortality rate was higher in patients with extended intervals (10–12 and ≥15 weeks). In this study, for adenocarcinoma of the esophagogastric junction, the percentage of patients who achieved a pCR was 15%–17% with an interval of 6–9 weeks, which was similar to the results of our study. In terms of safety, the incidence of pleural effusion increased significantly, which might be related to tissue edema caused by radiotherapy. The incidence of pulmonary infection in the perioperative period also significantly increased, and one patient died due to pulmonary infection. Therefore, lung function and the respiratory system should be fully evaluated in patients who receive nCRT before surgery. Lung function should be examined early after surgery to anticipate early detection and early treatment. Precise nutritional therapy for the perioperative period can improve postoperative complications (9).

Based on the successful experience of preoperative concurrent chemoradiotherapy for esophageal adenocarcinoma (17), the clinical possibilities of nCRT for AEG (14, 18) are endless, and the CROSS and POET trials (5, 6, 19, 20) confirmed the effect of nCRT on reducing recurrence and metastasis and improving survival and quality of life in AEG patients. In this study, neoadjuvant concurrent chemoradiotherapy significantly improved OS, especially for patients with local recurrence. The addition of radiotherapy is one of the main reasons for the decrease in the local recurrence rate. The most frequent type of distant metastasis in the two groups was peritoneal metastasis, which may be related to the fact that the inclusion criteria did not require a cytological examination of abdominal exfoliation. In addition, it is worth noting that some studies (21, 22) showed that HER-2 overexpression suggested a poor prognosis. In the subgroup analysis of this study, we also found that DFS and OS were significantly different between patients in the treated group with HER-2 overexpression and those with negative HER-2 expression, providing insights into our subsequent in-depth study. We have provided different targeted therapies for patients with different HER-2 expression statuses based on nCRT, and we expect reports on the effectiveness and safety of this trial in the future.

At present, nCRT is effective and relatively safe for patients with locally advanced Siewert type II and III AEGs and can be used as a standard treatment mode.

## Data Availability Statement

The raw data supporting the conclusions of this article will be made available by the authors, without undue reservation.

## Ethics Statement

The studies involving human participants were reviewed and approved by the Ethical Review Committee of Hebei Medical University. The patients/participants provided their written informed consent to participate in this study.

## Author Contributions

QZ designed the research. QZ, YT, JW, JZ, and XQ collected clinical data and performed the research. YL, LF, PY, ZZ, XZ, DW and BT performed the experiments. BT and YT analyzed the data. QZ and YT wrote the paper. All authors contributed to the article and approved the submitted version.

## Funding

This study was supported by the University Research Project of Hebei Province (grant no. ZD2019139) and the Medical Research Project of Hebei Province (grant no. 20201137).

## Conflict of Interest

The authors declare that the research was conducted in the absence of any commercial or financial relationships that could be construed as a potential conflict of interest.

## Publisher’s Note

All claims expressed in this article are solely those of the authors and do not necessarily represent those of their affiliated organizations, or those of the publisher, the editors and the reviewers. Any product that may be evaluated in this article, or claim that may be made by its manufacturer, is not guaranteed or endorsed by the publisher.
